# Development of Whole-Grain Rice Lines Exhibiting Low and Intermediate Glycemic Index with Decreased Amylose Content

**DOI:** 10.3390/foods13223627

**Published:** 2024-11-14

**Authors:** Ekawat Chaichoompu, Siriphat Ruengphayak, Siriluck Wattanavanitchakorn, Rungtiwa Wansuksri, Usa Yonkoksung, Phim On Suklaew, Sunee Chotineeranat, Sujitta Raungrusmee, Apichart Vanavichit, Theerayut Toojinda, Wintai Kamolsukyeunyong

**Affiliations:** 1 Rice Science Center, Kasetsart University, Kamphangsaen, Nakhon Pathom 73140, Thailand; ekawatun@gmail.com (E.C.); phatsterster@gmail.com (S.R.); siriluck.wat@hotmail.com (S.W.); vanavichit@gmail.com (A.V.); theerayuttoojinda638@gmail.com (T.T.); 2 Interdisciplinary Graduate Program in Genetic Engineering and Bioinformatics, Kasetsart University, Chatuchak, Bangkok 10900, Thailand; 3 National Center for Genetic Engineering and Biotechnology (BIOTEC), 113 Thailand Science Park, Phahonyothin Road, Pathum Thani 12120, Thailand; rungtiwa.wan@biotec.or.th (R.W.); usa.yon@ncr.nstda.or.th (U.Y.); sunee@biotec.or.th (S.C.); 4 Department of Home Economics, Faculty of Agriculture, Kasetsart University, Bangkhen, Bangkok 10900, Thailand; phimon.s@ku.th (P.O.S.); agrstrm@ku.ac.th (S.R.)

**Keywords:** glycemic index, amylose content, gelatinization temperature, rice breeding, soluble dietary fiber, insoluble dietary fiber

## Abstract

The demand for rice varieties with lower amylose content (AC) is increasing in Southeast Asia, primarily due to their desirable texture and cooking qualities. This study presents the development of whole-grain rice lines with low to intermediate glycemic index (GI) and reduced AC. We selected six rice lines for in vivo GI assessment based on their starch properties. We successfully identified two lines with low AC that exhibited low and intermediate GI values, respectively. Our findings indicate that dietary fiber (DF) content may significantly influence rice GI. The selected whole-grain low-GI line showed a higher ratio of soluble dietary fiber (SDF) to insoluble dietary fiber (IDF) compared to control varieties, highlighting SDF’s potential positive role in lowering whole-grain rice’s GI. This study underscores the feasibility of developing rice varieties with desirable agronomic traits, nutritional traits, and culinary attributes, particularly for individuals managing their blood sugar levels. Additionally, we proposed the positive role of starch composition, especially DF content, in modulating the GI of rice. This study reinforces the importance of incorporating starch properties and DF content into rice breeding programs to produce more health-oriented and marketable rice varieties.

## 1. Introduction

In recent years, the development of rice varieties with a low glycemic index (GI) has garnered significant attention, primarily due to the increasing prevalence of diabetes and the recognized health benefits associated with consuming low-GI foods. The GI system categorizes carbohydrates in various foods based on their effect on blood sugar levels after eating [1]. Foods with a low GI, typically defined as having a score of 55 or lower, are digested and absorbed more slowly, leading to a gradual rise in blood glucose levels. This characteristic is particularly beneficial for people with diabetes, as it assists in managing blood sugar levels and preventing sharp spikes. It also supports individuals looking to maintain a healthy weight [2,3]. Given that rice is a dietary staple for billions around the globe, creating low-GI rice varieties is essential [4]. Pursuing healthier staple food options that effectively control glucose levels has driven research and innovation in developing low-GI rice [5].

Several factors influence rice’s GI, with amylose content (AC) being critical [6]. Amylose is a type of starch comprising long, unbranched chains of glucose molecules, while amylopectin contains branched structures. The ratio of amylose to amylopectin in rice is crucial for determining its digestibility and the rate at which it raises blood glucose levels [7]. Research shows that rice varieties with high AC (usually over 25%) tend to have a lower GI [8]. This correlation is due to the structural characteristics of amylose, which render it less accessible to digestive enzymes, leading to slower digestion and absorption of carbohydrates [9]. As a result, blood glucose levels rise more gradually. In contrast, rice varieties with lower AC (typically below 20%) tend to have higher GIs. The high levels of amylopectin in these varieties promote faster digestion and a quicker release of glucose into the bloodstream, resulting in a more immediate glycemic response [10]. Numerous studies have confirmed that the relative amounts of amylose and amylopectin significantly affect the glycemic response, with higher AC generally associated with a lower GI [11].

Dietary fiber (DF), an indigestible carbohydrate, is vital in influencing the glycemic response to carbohydrate intake [12]. DF is categorized into soluble dietary fiber (SDF) and insoluble dietary fiber (IDF). SDF dissolves in water, creating a gel-like substance that can slow digestion, while IDF adds bulk to stool and supports overall digestive health [13]. By delaying glucose absorption, SDF effectively reduces the glycemic index of foods. When included in rice, SDF can help mitigate the rapid spikes in blood sugar typically associated with high-carbohydrate foods. Studies have indicated that rice varieties with higher DF content, particularly SDF, generally exhibit lower GIs [14]. For instance, whole-grain rice contains more fiber than white rice, aiding slower digestion and carbohydrate absorption [15].

Moreover, the preparation methods used for rice can significantly influence its fiber content and GI [16]. Overcooking rice can lead to the degradation of fiber structures, which may increase the GI. In contrast, less processed rice varieties are likely to preserve higher fiber levels, resulting in a lower GI. High-fiber rice varieties consistently show lower GI values than those with less fiber content [17]. A recent study found a strong connection between the ratio of SDF to IDF and the texture of cooked whole-grain rice [18]. Specifically, a greater SDF-to-IDF ratio was associated with softer rice that was less chewy and gummy. The research also suggested that this SDF-to-IDF ratio could be a potential biomarker for breeding softer and more appealing whole-grain rice.

Efforts to develop low-GI rice varieties have utilized traditional breeding techniques and advanced molecular methods. Conventional breeding involves crossing high-GI and low-GI rice varieties to produce offspring that inherit desirable traits, such as a lower GI, from their parent lines, leveraging the genetic diversity within existing rice varieties [19]. In contrast, molecular breeding employs sophisticated molecular markers to identify and select genes tied to low-GI traits, streamlining the development of varieties with targeted characteristics [20]. Techniques like marker-assisted selection (MAS) have proven effective in accelerating this process, leading to more efficient breeding programs [21]. Researchers are also investigating genetic modification to alter rice’s starch composition and digestion rates, reducing its GI [22]. The preparation methods for cooking rice can also significantly influence its glycemic impact, with studies examining how different techniques—such as parboiling, soaking, and various cooking methods—affect the glycemic response of rice varieties [16,23,24]. These findings indicate the complexity of factors affecting rice’s GI and highlight the need for a comprehensive approach to developing low-GI rice varieties that cater to diverse dietary needs and preferences.

Developing low-GI rice varieties is essential for tackling global health issues associated with diabetes and promoting healthier dietary choices. Recently, Ruengphayak et al. [25] employed a pseudo-backcrossing scheme to rapidly integrate multiple traits into the desirable genetic background of the aromatic rice variety ‘PinK3’, which is intolerant to flash flooding (Sub), bacterial leaf blight (BB), leaf/neck blast (BL), and the brown planthopper (BPH). The resulting rice varieties, named ‘PK+4’, demonstrate resistance to BB, BL, BPH, and Sub compared to PinK3, along with substantial grain yield enhancements ranging from 21% to 68% over the original varieties. Notably, research showed that the glycemic index of the PK+4#20A09 variety was measured at 48.1 [26], classifying it as low-GI, while its AC stands at 27.6%, categorizing it as high-amylose. 

The demand for rice varieties with lower AC is rising due to their softer texture and superior cooking qualities, with low-GI rice becoming increasingly sought after for its health benefits, especially for individuals managing blood sugar levels. The PK+4#20A09 variety already possesses favorable characteristics such as aroma, high yield, and pest resistance, making it an excellent foundation for further enhancements. The objective of this study is to develop a new rice variety based on the genetic background of PK+4#20A09, concentrating on decreasing AC to achieve a softer texture and enhanced cooking qualities while ensuring the variety maintains a low to intermediate GI to provide health benefits for individuals managing blood sugar levels.

## 2. Materials and Methods

### 2.1. Plant Materials and Breeding Scheme

**Figure 1:**
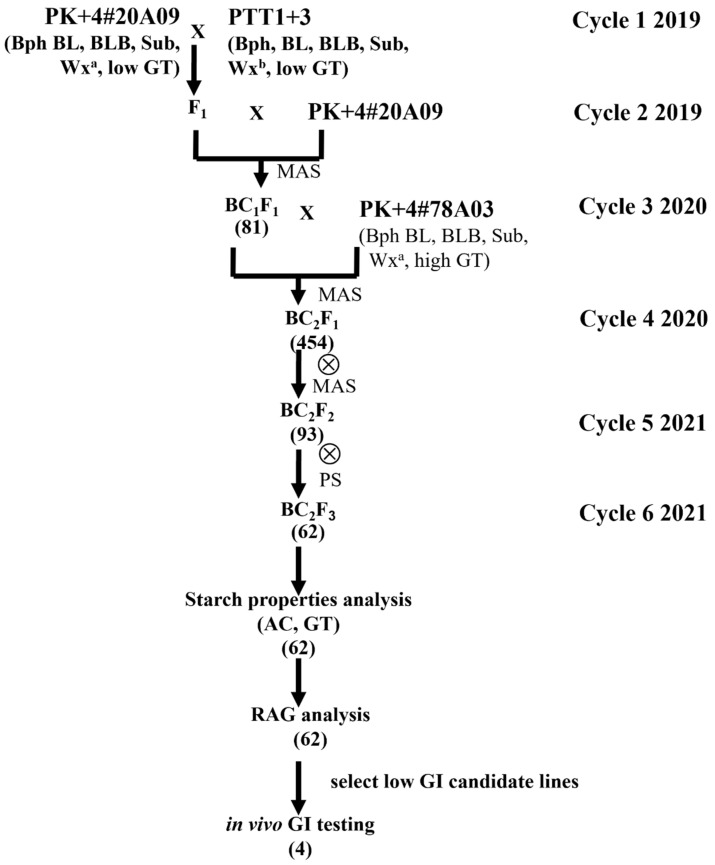
The breeding scheme generated the low-AC progeny of PK+4#20A09. The symbol ⊗ means self-pollination.

PK+4#20A09 is an aromatic rice cultivar with high yield, irrigation and flash flooding (Sub) tolerance, and resistance to bacterial leaf blight (BB), leaf-neck blast (BL), and the brown planthopper (BPH) [25]. In this study, the low-AC trait was introduced to PK+4#20A09 from the improved Pathum Thani 1 (PTT1) [27], known as PTT1+3 (RGD16001-93-90-2), developed by the Innovative Plant Biotechnology and Precision Agriculture Research Team (APBT), National Center for Genetic Engineering and Biotechnology (BIOTEC), Thailand. PTT1+3 was crossed with PK+4#20A09 to produce the F_1_ generation (Figure 1). The F_1_ generation was then backcrossed with PK+4#20A09 to generate the BC_1_F_1_ generation, selecting plants with desirable traits such as resistance to BB, BPH, BL, and Sub by linked markers. The selected BC_1_F_1_ plants were further backcrossed with PK+4#78A03 to create the BC_2_F_1_ generation, with continued selection for the desired traits with linked markers. The selected plants were self-pollinated to form the BC_2_F_2_ generation, ensuring homozygous genes for the desired characteristics. This process continued with another round of self-pollination to establish the BC_2_F_3_ generation, focusing on selection by linked markers for attributes such as gelatinization temperature (GT) and AC. The yield traits of the BC_2_F_3_ population were collected during the 2022 dry season at the Kasetsart University Kamphaeng Saen Field Station, including flowering dates, days to maturity, plant heights, tiller numbers, and weights. The plot size was 2.50 × 2.50 m.

### 2.2. Starch Property Analysis

The AC of candidate rice lines was measured using the colorimetric method [28]. The GT was measured using the alkali test [29]. The pasting properties were studied using the Rapid Visco Analyzer (RVA). This analysis measures changes in the viscosity of starch slurry during heating and cooling. It provides crucial information about rice’s cooking and eating quality as peak viscosity, the maximum viscosity reached during heating; trough viscosity, the minimum viscosity reached after the peak; final viscosity measured after cooling; breakdown, the difference between peak and trough viscosity; and setback, the difference between final and trough viscosity, respectively. The in vitro starch digestion employed an in vitro method [30] to simulate starch digestion in the human digestive system. This analysis measured the glucose released from cooked rice at different time points. The rapidly available glucose (RAG) is released at 20 min, and the slowly available glucose (SAG) is released at 120 min. Finally, the in vitro kinetic starch digestion data were used to estimate the GI [31].

#### 2.2.1. Starch and Amylose Determination

The starch content of rice flour was measured according to the AOAC standard method 996.11 using a Megazyme Assay Kit (Megazyme International, Wicklow, Ireland). The apparent AC of the rice was determined using the iodine colorimetric method. The standard was potato amylose type II (Sigma–Aldrich, St. Louis, MO, USA).

#### 2.2.2. Alkali Spreading Value (ASV) and Gelatinization Temperature (GT)

Alkali spreading value (ASV) serves as a measure of GT and exhibits an inverse relationship with GT. The ASV method involves incubating six milled rice grains in 100 mL of 1.7% KOH at 30 °C for 23 h [29]. It quantifies the degree of spreading using a seven-point scale (ranging from 1 = intact to 7 = greatly dispersed) and corresponds to GT as follows: 1–2, high (74.5–80 °C); 3, high–intermediate; 4–5, intermediate (70–74 °C); and 6–7, low (<70 °C).

#### 2.2.3. Paste Properties

Paste properties of the starch sample (9.3% dwb in distilled water) were evaluated using a Rapid Visco Analyzer (RVA4, Newport Scientific, Warriewood, NSW, Australia). The paddle speed was set at 960 rpm for the first 10s and 160 rpm for the rest of the analysis. The suspension was heated from 30° to 95 °C at a rate of 5 °C/min and held at 95 °C for 6 min. The paste was then cooled down to 50 °C at a rate of 5 °C/min and held at this temperature for 10 min until the end of the experiment. Pasting profiles were determined in duplicate, and the evaluated parameters were averaged.

#### 2.2.4. Starch Digestibility and In Vitro Starch Digestion Kinetics

The starch digestibility assessment followed the protocol developed by Goni et al. [30]. A digestion test assessed the digestibility of five rice varieties: RD 43, M7881, Basmati, PK+4#20A09, and PK+4#78A03 and selected lines. These varieties were subjected to two distinct sample preparation processes: freeze-dried fine powder (milled into powder and stored in an airtight container at room temperature until further analysis) and cooked whole-grain rice cut to approximately 0.3 mm in size. The two distinct prepared samples were soaked in water (using a 1:2 ratio) and then cooked in a stainless-steel steamer for 30–40 min until no whitish core remained. To determine the digestibility of cooked rice, pepsin powder (P7000, Sigma–Aldrich, St. Louis, MO, USA) (10% *w*/*v*) was added into 0.05 M HCl–KCl buffer (pH 1.5) to obtain 250 units/mg solid. The pepsin solution (2 mL) was added to a flask containing cooked rice powder (50 mg) and HCl–KCl buffer (5 mL). The sample solution was then incubated in a 40 °C water bath for 60 min with constant shaking. The pH of the sample solution was adjusted to 6.9 using 0.05 M Tris maleate buffer, added with alpha-amylase (porcine pancreatic) (Megazyme International, Wicklow, Ireland) (2.5 mL), and incubated in a 37 °C water bath with constant shaking. Sample aliquots were withdrawn at 20, 30, 60, 90, 120, 150, and 180 min to determine available glucose content using a D-glucose assay kit (K-GLUC, Megazyme International, Wicklow, Ireland). Rapidly available glucose (RAG) was obtained as the glucose released after 20 min, and the slowly available glucose (SAG) was obtained as the glucose released after the further 100 min incubation. Finally, the in vitro starch digestion kinetics were used to estimate the glycemic index (GI). The GI of the samples was then calculated using the following equation: GI = 39.21 + 0.803(H90) [30].

#### 2.2.5. Proximate Analysis of Whole-Grain Rice

Rice varieties/lines used for proximate analysis comprised 2 PK+4 varieties, PK+4#20A09 and PK+4#78A03, and 4 BC2F3 progenies, including 6D11, 9D02, 12A05, and 2G04. Whole-grain rice samples were coarsely ground with a blender, followed by fine grinding and screening into particle sizes of 200 μm using a speed rotor mill, Pulverisette 14, Fritsch. The flour was stored in an airtight container at −20 °C until required for proximate analysis. The gravimetric method measured the moisture content based on the ISO method 712:2009 [32]. The total fat was determined using Soxhlet extraction with petroleum ether based on AOAC method 945.16 [33]. Kjeldahl analysis determined crude protein according to the AOAC method 2001.11 [34]. Crude ash was determined by incineration at 525 °C for five hours according to AOAC method 942.05 [35]. DF was determined by enzymatic gravimetry combined with high-performance liquid chromatography (HPLC) based on a modification of AOAC methods 991.43 and 985.29 and AOAC methods 2009.01 and 2011.25 [33]. All whole-grain rice samples were soaked in water in a ratio of 1:2 in aluminum cups and then cooked in a stainless-steel steamer for 30–40 min until no white starch core could be observed before the analysis. To determine the moisture content in cooked rice, the samples were dried in an air oven at 60 °C for 3 h, followed by 105 °C for 16 h, and then weighed.

#### 2.2.6. Dietary Fiber Content Analysis

DF was determined by measuring carbohydrates with a degree of polymerization of more than 2 (DP > 2), which are not hydrolyzed by the endogenous enzyme in the small intestines of humans. The enzymatic method based on the Official Methods of Analysis of AOAC International (AOAC) methods 991.43 and 985.29 (K-TDFR, Megazyme International, Wicklow, Ireland) was used to estimate the DF content of whole-grain rice samples in this study. The sample was subjected to sequential enzymatic digestion by heat-stable α-amylase, protease, and amyloglucosidase. The solution was filtered after complete digestion to separate the insoluble (residue) and soluble (filtrate) fractions. The weight of the residue corrected for crude protein and ash formed the total quantity of IDF, which was calculated as the percentage of whole-grain rice flour. For deionization, the filtrate was further passed through a column packed with mixed-bed ion exchange resin, following which the deionized solution was concentrated and filtered again through a 0.45 μm membrane filter. The filtrate consisting of the SDF was quantified by high-performance liquid chromatography with a refractive index detector (Shimadzu RID-10A HPLC system, Shimadzu Corporation, Kyoto, Japan) based on a modification of Ohkuma’s method and AOAC methods 2009.01 and 2011.25 (K-INTDF, Megazyme International, Wicklow, Ireland) [33]. SDF was expressed as the percentage of whole-grain rice flour, whereas total DF (TDF) comprised the sum of IDF and SDF.

#### 2.2.7. Statistical Analysis

The analysis was performed using Statgraphics Centurion XVII software (version 16.1.11) (Statpoint Technologies, Warrenton, VA, USA). The data were analyzed by one-way analysis of variance and using Duncan’s multiple range test to determine statistically significant differences among the samples. Significant differences are indicated by different letters in rows when the *p*-value is lower or equal to 0.05.

### 2.3. In Vivo Glycemic Index (GI) Analysis

#### 2.3.1. Experimental Design

The research followed the methods established by Wolever et al. [36]. Twelve participants consumed a reference food (glucose) and six test foods, consisting of cooked whole-grain rice varieties/lines (Supplementary Figure S1), each containing 50 g of available carbohydrates. The participants included eleven females and one male, with a mean age of 39.00 ± 9.98 years, a Body Mass Index (BMI) of 20.78 ± 1.27 kg/m^2^, and a fasting blood glucose level of 90.80 ± 4.76 mg/dL. All participants were asked to refrain from using medications or supplements that could affect blood glucose levels and maintain their usual dietary intake and physical activity before and during this study. They provided written informed consent before enrolling in this study. The Kasetsart University Research Ethics Committee approved the study protocol of the Kasetsart University, Bangken (KUREC-HSR66/043), per the Declaration of Helsinki.

The GI was determined from blood sugar levels measured through capillary blood samples collected via finger pricks post-consumption [37]. This study was conducted over eight weeks, including a wash-out interval of two days [37,38]. The GI for each rice variety/line was evaluated using an online random selection tool (https://www.randomlists.com/team-generator, accessed on 13 November 2024. Participants were randomly assigned to receive a specific test food, and during subsequent sessions, a different test food was given, ensuring that all six varieties/lines were tested without repetition. Blood sugar levels were also measured with the reference food before and after the study sessions. Consequently, each participant was required to complete a total of eight sessions, with each session lasting approximately 2.5 h from the first to the seventh blood draw following the consumption of the provided meal. Participants were allowed to drink up to 300 mL of water.

Participants were instructed to fast for 10 to 12 h before participation, beginning at 9:00 PM the night before the blood draw scheduled for 7:00 AM at the Faculty of Agriculture, Kasetsart University, Thailand. As outlined in Table 1, the test foods and the reference food were prepared for participants from 7:00 AM to 9:00 AM. Participants were required to consume the provided meal within 15 min. Blood sugar levels were measured from capillary blood samples at seven intervals: before eating (0 min) and then at 15, 30, 45, 60, 90, and 120 min after consumption. Throughout this study, participants were able to maintain their regular daily activities.

#### 2.3.2. Reference and Test Foods

The reference food utilized in this study was glucose, administered at a dose of 50 g dissolved in 250 mL of plain water, which participants drank. The test foods included six varieties/lines of whole-grain rice: PK+4#20A09, PK+4#78A03, 6D11, 9D02, 12A05, and 2G04. The rice samples were cooked using a DIGITAL TOSHIBA rice cooker (RC-5MMWTA, Tokyo, Japan) with a rice-to-water ratio of 1:2 and cooked for 30 to 40 min before immediately being served to participants. 

#### 2.3.3. Measuring Blood Sugar Levels, GI Calculation, and Statistical Analysis

Blood sampling involved 56 finger prick draws in this study. For each collection, capillary blood was obtained from participants after the puncture site was disinfected with a 70% alcohol pad. Blood was collected by pricking the middle of the ring or middle finger, avoiding the pinky, thumb, and swollen fingers (https://www.sarstedt.com/en/products/new-products/tip-of-the-month-capillary-blood-collection/, accessed on 13 November 2024. The second drop of blood was utilized for measurement [39]. Following the blood draw, the punctured area was dried and pressed with sterile cotton until the bleeding stopped. Blood samples were analyzed using the Accu-Chek^®^ Active glucometer (Roche Diagnostics GmbH, Mannheim, Germany) at the Faculty of Agriculture, Kasetsart University, Thailand. Participants waited in a designated room between blood draws, where clearly defined areas for sampling and waiting were established. Tables, chairs, and power outlets were provided to ensure volunteers’ comfort while waiting for blood draws. After each session, all participants were offered milk and bread.

All information related to participants was kept confidential. Research findings were disclosed only in summary form, using participant identification numbers for reference. Furthermore, blood samples were not collected from participants for any experiments beyond this study. Upon completion of the research, all blood samples and any derived components were destroyed.

The GI of the six rice varieties/lines was calculated using Wolever’s formula [40]. The IAUC of post-meal blood sugar levels was calculated using the trapezoidal rule with SigmaPlot 14 Software [41]. Data analysis was conducted using SPSS Statistics Version 26, with results presented as mean ± standard deviation (mean ± SD). A paired sample *t*-test was used to analyze differences between the test foods and the reference food, with a significance level set at 0.05.

## 3. Results

### 3.1. Development of the Low-Amylose-Content Progeny of PK+4#20A09

The development process began with crossbreeding between PTT1+3 (male parent) and PK+4#20A09 (female parent), which produced 46 F_1_ plants. In cycle 2, these F_1_ plants were backcrossed with the recurrent parent, PK+4#20A09, yielding 500 BC_1_F_1_ seeds. Through targeted marker-assisted selection (MAS), 81 fully heterozygous BC_1_F_1_ plants with BL and Wx traits were selected. The genes Sub, BPH, and BB conferred resistance, as both the male and female parents possessed these genes.

In cycle 3, the recurrent parent was changed to PK+4#78A03, a variety from the same parental lines that exhibited superior BL resistance, no symptoms of dirty panicle disease, and a higher GT. This backcross yielded 2162 BC_2_F_1_ seeds. Genotyping results indicated that 454 fully heterozygous BC_2_F_1_ plants with BL, Wx, and GT traits were selected and self-pollinated in cycle 4 to generate BC_2_F_2_ seeds.

Cycle 5 involved cultivating BC_2_F_2_ plants from 50 families, with 32 plants per family, to establish stable lines. Full-target MAS identified 93 BC_2_F_2_ plants with homozygous resistance genes (Table 2). These progenies were classified into four groups based on starch properties: Group 1—high AC and high GT; Group 2—low AC and high GT; Group 3—high AC and low GT; and Group 4—low AC and low GT. All BC_2_F_2_ progeny carried favorable homozygous alleles for the resistance genes/QTLs, including Sub, BB (xa5 and Xa21), Bph3, Bph32, and qBL1-qBL11.

In cycle 6, BC_2_F_3_ seeds obtained from the self-pollination of selected BC_2_F_2_ plants were used for observation planting. This observation planting included a population of 62 lines to assess yield performance. The observation results for the BC_2_F_3_ population provided insight into flowering dates, days to maturity, and yields (see Supplementary Table S1 and Supplementary Figure S2). Lines 5D03 and 8B12 exhibited the most prolonged flowering periods at 135 days, whereas line 5G06 had the shortest at 99 days. For maturity, line 5B06 had the shortest period at 127 days, while line 5D03 took the longest at 158 days. Regarding yield, line 10D04 recorded the highest rice weight, followed by lines 15B12 and 14H08 (with 4335, 4271, and 3959 kg per hectare, respectively). Conversely, line 4E05 showed the lowest yield, producing only 348 kg per hectare. Finally, the BC_2_F_4_ seeds were collected for further starch properties, RAG and SAG testing, and in vitro and in vivo GI analysis.

### 3.2. Amylose Content and Alkali Spreading Value Analysis

**Figure 2:**
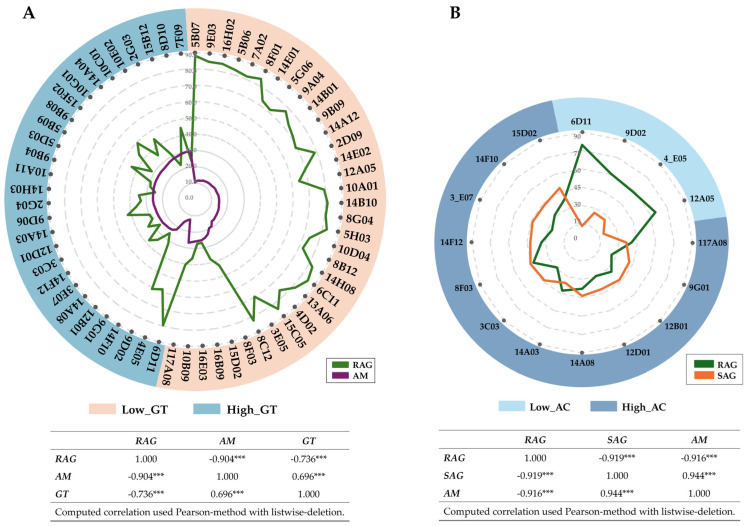
The relationship between RAG values and AC (**A**) and between AC and RAG values and SAG (**B**) in whole−grain rice. *** indicate statistically significant correlation at *p* ≤ 0.001.

The selection of the samples from the BC_2_F_3_ generation population, based on AC and alkali spreading value (ASV), serves as a measure of GT as criteria for selection, resulting in 34 lines in the low-GT group and 29 lines in the high-GT group. The AC in the low-GT group ranged from 10.52% to 26.80%, while in the high-GT group, it ranged from 12.57% to 29.74%. The RAG values for the low-GT group ranged from 26.83 to 87.95; for the high-GT group, the RAG values ranged from 22.92 to 79.57. A clear relationship was observed, showing that rice with higher AC tends to have lower RAG values in both the low- and high-GT groups, as shown in Figure 2A and Supplementary Tables S2 and S3.

As a result, 16 rice lines were selected for further testing. Of these, four lines in the low-AC group (6D11, 9D02, 4E05, and 12A05) and 12 lines in the high-AC group (117A08, 9G01, 12B01, 12D01, 14A08, 14A03, 3C03, 8F03, 14F12, 3E07, 14F10, and 15D02) were selected for further SAG testing. The results showed that the four lines in the low-AC group had SAG values ranging from 13.5% to 26.2%, while the 12 lines in the high-AC group had SAG values ranging from 35.6% to 48.1%, as shown in Figure 2B and Supplementary Table S4. Pearson’s correlation analysis demonstrated a significant negative correlation between AM and RAG and a significant positive correlation between AM and SAG.

### 3.3. Starch Property

**Figure 3:**
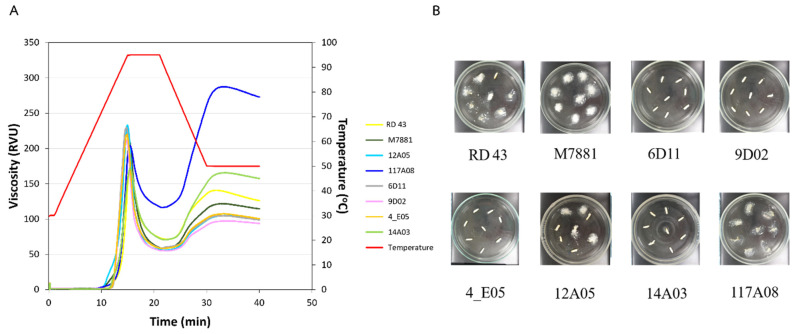
The paste properties of flour from various rice varieties/lines in a 30 mM silver nitrate solution were determined using a Rapid Visco Analyzer (**A**) and alkali spreading (**B**).

Based on AC, GT, and starch digestibility results (Supplementary Table S4), six rice lines were selected, including 4_E05, 6D11, 9D02, 12A05, 14A03, and PK+4#117A08 as the control variety. The lines classified as high−GT and low−AC include 6D11, 9D02, and 4_E05, while the line with low GT and low AC is 12A05. Line number 14A03 has high GT and high AC. The variety PK+4#117A08 is classified as low−GT and high−AC. In addition, these variety/line RAG values range from 40.3 in PK+4#117A08 to 79.6 in line 6D11. These varieties/lines were further analyzed for starch content and viscosity. In addition to these six lines, two rice varieties with low and high GI, RD43 and M7881, were included as controls for comparison. The results showed that the tested varieties/lines and control varieties had similar starch content, ranging from 76.45% to 83.26% for the tested lines and being 77.20% for RD43 and 82.40% for M7881, the control varieties (Table 3). The pasting temperature of the tested lines in the low−GT group was close to that of the control varieties. In contrast, the tested lines in the high−GT group exhibited a higher pasting temperature than the controls. When comparing the final viscosity and setback from the trough, the control varieties had values of 115 and 56 RVU for M7881 and 126 and 55 for RD43, while the tested lines ranged from 94 to 273 RVU and 39 to 157 RVU, as shown in Figure 3A and Table 3. The ASV analysis of eight rice varieties/lines indicated that 6D11, 9D02, 4_E05, and 14A03 were classified as high−GT with an ASV scale of 2. In contrast, 12A05 and RD43 were classified as intermediate GT, with ASV scales of 4 and 5, respectively. M7881 and 117A08 were categorized as low−GT, with an ASV scale of 6 (Figure 3B).

### 3.4. In Vitro Analysis of Starch Digestion Kinetics

**Figure 4:**
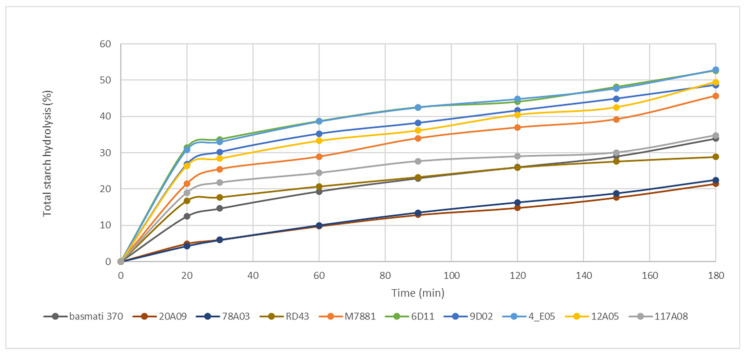
The rate of starch digestion by amylase enzyme over varying incubation times (20 to 180 min) differs among cooked whole−grain rice varieties. The samples were prepared as cooked rice pieces cut to 0.3 mm in size.

The digestion of cooked whole−grain rice by amylase enzymes for 0 to 180 min with different rice particle sizes (fine powder and 0.3 mm particle size) revealed distinct hydrolysis curves depending on the rice variety and grain particle size. The hydrolysis curve of cooked rice with a particle size of 0.3 mm showed a continuous increase in digestible starch concentration over 0–180 min (Figure 4). This finding indicates that the grain particle size affects the efficiency of starch digestion by amylase enzymes. In contrast, the hydrolysis curve of the fine powder form of cooked rice exhibited a two−phase pattern. In the first phase (0–90 min), the concentration of digestible starch increased with time, while in the second phase (90–180 min), the digestion reached equilibrium (Supplementary Figure S3).

When fitting the hydrolysis curve using an exponential model and first−order equation, the equation used was C = Cα (1 − e^−kt^), where C represents the concentration of digestible starch at t time, Cα is the starch concentration at equilibrium, k is the kinetic constant, and t is the digestion time. This allowed for calculating k and Cα values for the cooked rice in fine powder form, as shown in Table 4. Upon examining the k and Cα values of the control rice varieties (RD 43, M7881, and PK+4#117A08) compared to the candidate lines (6D11, 9D02, 4_E05, and 12A05), it was observed that the line numbers 4_E05 and PK+4#117A08 had lower k constants, while other tested lines had k constants similar to those of the control varieties.

To calculate the in vitro GI, the equation GI = 39.21 + 0.803(H90) was used, where H90 represents the amount of digestible starch compared to the total starch in cooked rice. The calculated GI values for the cooked rice in fine powder form showed that the GI of the control rice varieties (RD 43, M7881, and PK+4#117A08) and candidate rice lines (6D11, 9D02, 4_E05, and 12A05) ranged between 108 and 116, categorizing them as high−GI. In contrast, as expected, the calculated GI values for the cooked rice grains with a size of 0.3 mm were 54 for RD 43, 72 for M7881, and 68 for PK+4#117A08, classifying RD 43 as low−GI, M7881 as high−GI, and PK+4#117A08 as intermediate−GI, respectively. The four tested rice lines were classified as high−GI (GI values ranging from 75 to 84). Notably, 12A05 has the lowest calculated GI value among the tested rice lines.

This analysis selected three rice lines, including 6D11, 9D02, and 12A05, with in vitro GI of 80, 79, and 75 for the further study of in vivo GI. We also included 2G04, the high-GT and high-AC rice lines, in the next in vivo GI analysis as the possible low-GI rice line (Supplementary Table S3). The parental varieties PK+4#20A09 and PK+4#78A03 were also included in the in vivo GI analysis.

### 3.5. Nutrition Analysis of Tested Rice Samples

All rice varieties/lines used in the in vivo GI testing have been analyzed nutritionally following the standards established by the Association of Official Analytical Chemists (AOAC), with the results shown in Table 5. The nutrition values of cooked whole-grain rice (per 50 g) revealed that 12A05 has the highest amount of fat (1.00 ± 0.03 g), total carbohydrates (29.50 ± 0.04 g), and DF (1.15 ± 0.04 g). In contrast, 9D02 has the highest amount of energy (131.71 ± 0.15 kcal), PK+4#78A03 has the highest amount of protein (2.78 ± 0.00 g), and 2G04 has the highest amount of available carbohydrates (27.99 ± 0.03 g). The portion of rice given to participants in each session guaranteed the consumption of 50 g of available carbohydrates. Nutritional information and data for each rice variety/line used in the in vivo GI testing are presented in Table 6. 

### 3.6. In Vivo GI Analysis of Cooked Whole-Grain Rice

**Figure 5:**
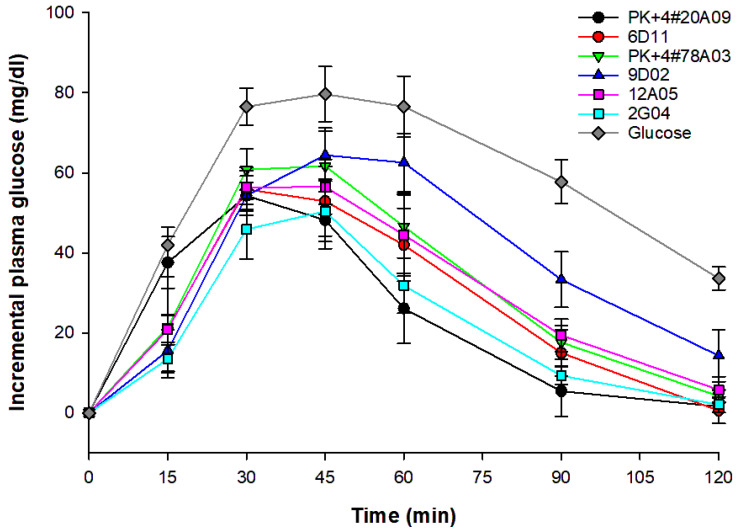
The postprandial blood glucose curves of six rice samples and reference food (glucose).

The in vivo GI values for six rice varieties/lines are shown in Table 7. The GIs for PK+4#20A09, PK+4#78A03, and 2G04, the high-AC rice varieties/lines, are 41.14 ± 10.97, 50.47 ± 10.29, and 41.79 ± 9.54, respectively, categorizing them as low-GI rice. In contrast, for the three low-AC rice lines tested, it was found that 6D11 (12.57% AC) falls into the intermediate-GI category with a GI of 56.11 ± 14.10, 12A05 (14.42% AC) falls into the low-GI category with a GI of 53.92 ± 14.87, and 9D02 is classified as a high-GI line with a GI of 74.35 ± 11.47. Notably, the GI values of all rice varieties/lines were significantly lower than those of the reference food (*p* < 0.001). In addition, Figure 5 illustrates the postprandial blood glucose curves of six rice varieties and a glucose reference. The high-AC varieties/lines (PK+4#20A09, PK+4#78A03, and 2G04) exhibited significantly lower and slower glucose responses compared to the reference glucose and the low-AC lines (6D11, 9D02, and 12A05). The low-AC lines showed faster and higher glucose increases, particularly 9D02, highlighting the impact of AC on the glycemic response. All rice varieties showed significantly lower glucose responses than the glucose reference.

### 3.7. Dietary Fiber Content

For the IDF and SDF test results, shown in Table 8, the SDF–IDF ratios of PK+4#20A09 and PK+4#78A03, the control varieties, are 0.10 and 0.25, respectively, showing that the SDF of PK+4#78A03 is higher than that of PK+4#20A09. Among the tested rice lines, line numbers 6D11 and 9D02 had SDF–IDF ratios of 0.18 and 0.08, respectively. These two lines had similar amounts of AC at 12.57 and 15.15%, classified as low-AC. On the other hand, line numbers 12A05 and 2G04 had higher SDF-to-IDF ratios at 0.33 for both lines. Interestingly, while 2G04 was classified as high-AC with 25.80%AC, 12A05 was classified as low-AC with 14.42%AC. Finally, it should be noted that 12A05 had the highest amount of SDF (1.15%) among the rice varieties/lines tested.

## 4. Discussion

Developing low-GI rice varieties is an essential research focus in light of the rising prevalence of diabetes and other health issues associated with high-glycemic-load foods. Our study adds to this crucial area by examining the starch composition, starch digestibility, DF, and in vivo GI of rice and demonstrating the use of MAS in creating new rice varieties with favorable traits. We highlight the significant role of starch composition, particularly AC, in influencing the GI of rice [8,9], noting that rice with higher AC generally exhibits a lower GI because amylose is digested more slowly than amylopectin. Our possible low-GI rice line, 2G04, was confirmed by the in vivo GI test to be categorized into the low-GI group as it is the high-AC rice. Additionally, we found the critical impact of DF, especially SDF, on the GI of rice [14,16]. Our findings indicate that increasing the ratio of SDF to IDF may significantly lower the GI, even independently of AC, underscoring the importance of DF composition in rice breeding for better health outcomes. The high-SDF–IDF-ratio rice line 12A05 showed an in vivo GI of 53.92 and a low AC of 14.42%. Furthermore, our in vivo GI testing with human volunteers provides a more accurate evaluation of rice’s effect on blood sugar levels than in vitro methods [23,31]. This research enhances the knowledge base for developing soft-texture rice varieties with lower GI, aligning with current trends emphasizing the critical roles of starch composition, DF, and MAS in rice breeding for improved health outcomes and sustainable food systems.

In this study, we used crossbreeding techniques to maintain beneficial traits such as resistance to both biotic and abiotic stresses from the PK+4 varieties while incorporating the advantageous softened texture (low AC) from PTT1+3. To enhance genetic improvements, we employed MAS as part of our breeding strategy [42]. We also changed the recurrent parent from PK+4#20A09 to PK+4#78A03 to strengthen the progeny’s resistance to dirty panicle disease and enhance grain traits [43]. Notably, the classification of BC_2_F_2_ progenies into four distinct groups based on starch properties is significant, given the rising consumer demand for high-quality rice (Table 2). Recent studies emphasize the critical role of starch composition in determining cooking properties and marketability [44]. Our attention to AC and grain traits aligns with consumer expectations and addresses evolving processing requirements, showcasing how breeding goals have adapted to market dynamics [45]. Additionally, traits such as flowering dates and days to maturity among BC_2_F_3_ progenies are essential for assessing the adaptability of new cultivars to different environmental conditions [46]. The integration of MAS and consideration of market-driven starch properties demonstrate a comprehensive understanding of the complexities and future directions of rice breeding [47].

When considering the relationship between AC and RAG (Figure 2), it is evident that AC exhibits a positive correlation with starch digestion. These findings are consistent with previous studies, demonstrating that medium amylose chains can effectively reduce the total digestible starch fraction [48,49]. This intriguing phenomenon can be attributed to the linear and flexible structure of amylose, which enables it to form complexes with lipids during cooking or retrogradation [50]. Recent research has further elucidated this process, revealing that retrograded starch with high AC is more resistant to digestion by amylase enzymes [51]. Moreover, a study by Li et al. reinforces this understanding by highlighting how the spatial arrangement of amylose and amylopectin molecules influences the gelatinization and retrogradation processes, thereby affecting the starch’s digestibility [52]. Research indicates that specific ratios of amylose to amylopectin in rice can enhance resistant starch formation, which has potential health benefits, such as improved glycemic control and increased satiety [53]. Another recent study by Xie et al. explored the impact of cooking methods on starch digestibility, finding that steaming rice leads to a lower glycemic response due to the preservation of amylose structure compared to boiling [54]. 

For the in vitro starch digestibility test of the rice lines identified as whole-grain low-GI candidates in comparison to control varieties, it was observed that the grain particle size (fine powder and 0.3 mm pieces) utilized in the digestibility tests significantly influenced starch digestibility (Table 4). Previous studies, such as those conducted by Al-Rabadi et al., have reported that particle size plays a crucial role in the digestive process, where smaller particles tend to have increased surface area and promote faster enzymatic breakdown [55]. Recent works emphasize the necessity of considering the physical form of rice and its cooking methods, as these factors can substantially alter starch digestibility and consequently affect glycemic responses [56,57]. These studies demonstrated that varying cooking techniques and preparation methods led to significant differences in the release of glucose during simulated digestion trials. Furthermore, additional studies, such as those conducted by Shen et al., have found that rice processing—mainly when it involves milling and cooking—can modify starch crystalline structures, thus impacting digestibility [58]. By ensuring that the testing methodologies accurately reflect real-world consumption, we can evaluate the nutritional profiles of different rice lines and develop low-GI varieties catering to health-conscious consumers.

In our study, rice varieties/lines identified as in vitro high-GI show significant disagreement in measured values compared to in vivo tests (Table 4 and Table 7), emphasizing the necessity for comprehensive GI testing [59]. Therefore, further research focused on accurate in vitro starch digestibility is vital for gaining deeper insights into the primary and contributing factors influencing the selection of low-GI candidate rice varieties. Recent studies have utilized near-infrared spectroscopy (NIRS) to more accurately predict starch digestibility, enabling faster screening of potential low-GI candidates [60]. Additionally, understanding starches’ gelatinization and retrogradation behaviors under diverse cooking conditions can facilitate the development of rice varieties that meet low-GI standards and preserve superior cooking qualities, addressing both health considerations and consumer preferences in the contemporary food market [61].

Cooking and eating quality (CEQ) is intricately linked to the apparent viscosity, which measures GT and paste properties analyzed using an RVA [62]. Recent research revealed the importance of these parameters in determining the overall acceptability of various rice varieties, as they directly influence texture and mouthfeel. Wattanavanitchakorn et al. found a strong correlation between the ratio of SDF to IDF and the texture of cooked whole-grain rice, suggesting that adjusting this ratio could enhance CEQ [18]. Studies have shown that higher levels of SDF improve texture and lower the glycemic index (GI), thereby enhancing the nutritional profile of rice [12]. 

The postprandial blood glucose levels for six rice samples were evaluated by calculating the ratio of the areas under their hydrolysis curves relative to a glucose reference (Figure 5). While the hydrolysis curves were generally similar across samples, significant differences among rice varieties/lines were identified. Varieties/lines with low AC exhibited faster hydrolysis during the initial 30 min compared to those with high AC, with hydrolysis rates continuing to rise, peaking at 45 min before declining towards 120 min. The RAG is indicated by glucose release after 20 min, while the SAG reflects glucose release from 20 to 120 min, suggesting a slower, more complete digestion process [63]. This research highlights a complex relationship among AC, DF, and GI, consistent with various studies emphasizing the importance of amylose in influencing GI [6,59,64]. The differing starch proportions—particularly the amylose to amylopectin ratio—significantly impact the GI among rice varieties [65]. High-amylose varieties/lines (e.g., PK+4#20A09, PK+4#78A03, 2G04) consistently displayed lower GI values, reinforcing the relationship between elevated amylose and decreased glucose absorption [66,67]. Additionally, this study illuminated the role of DF, especially the ratio of SDF to IDF. For instance, line 12A05, despite having low AC, demonstrated a low GI likely due to its high SDF–IDF ratio, correlating with findings that SDF reduces glucose absorption and, consequently, GI [12,68]. Furthermore, DF intake has been linked to reductions in glucose, insulin, and serum lipid levels in both diabetic and non-diabetic individuals [69,70]. In contrast, line 9D02, classified as high-GI, had a similar AC to 12A05 but a substantially lower SDF–IDF ratio. The intermediate GI value of line 6D11 highlights the interactive roles of AC and DF in determining glycemic responses. Overall, these findings underscore the impact of starch composition and fiber content on the GI of rice. 

The ratio of SDF to IDF significantly impacts the GI value of rice, which is crucial for understanding its effect on blood sugar regulation [12]. A higher SDF–IDF ratio may lead to a lower GI value because soluble fibers can slow down glucose absorption in the intestines, promoting better glycemic control [71]. This phenomenon is due to the capacity of SDF to create a gel-like consistency in the digestive tract, which slows down the enzymatic breakdown of carbohydrates [72]. Conversely, a higher IDF content can increase the GI value, as insoluble fibers aid in the bulk of stool formation but do not significantly influence glucose absorption [73]. Therefore, optimizing the SDF-to-IDF ratio in rice through breeding or processing methods can enhance its nutritional profile, making it more suitable for individuals managing diabetes or aiming for stable blood sugar levels. Further research is essential to quantify these effects and establish guidelines for rice consumption that consider varying SDF–IDF ratios.

Basmati rice is well known for its CEQ and is known to have a low to intermediate GI [26,74]. While Basmati varieties are categorized as having intermediate amylose content [75], our newly developed rice lines possess even lower amylose levels, contributing to a softer texture that gained popularity in Southeast Asia. Significantly, research has not explored the SDF–IDF ratio in the Basmati varieties. Investigating this ratio is essential for understanding its potential role in lowering the GI and uncovering the mechanisms that could lead to reduced GI in rice varieties. 

## 5. Conclusions

This study successfully developed new whole-grain low-AC rice lines with a low and intermediate GI derived from the PK+4#20A09 genetic background, focusing on reducing AC while maintaining desirable low-GI traits. We identified four promising rice lines by analyzing the starch properties and in vitro GI. The in vivo GI testing, conducted with human volunteers, identified two rice lines classified as low-GI, including 12A05 and 2G04, which have AC of 14.42 and 25.80%, respectively. Moreover, one line, 6D11, with 12.57% AC, was found to be intermediate-GI. This study revealed the possible role of DF composition, specifically the ratio of SDF to IDF, in lowering the GI of rice. The analysis of DF content in the selected low-GI lines revealed that they had higher ratios of SDF to IDF compared to the high-GI varieties/lines, indicating that SDF may significantly contribute to their lower GI. These findings underscore the importance of considering starch properties and DF content in rice breeding to develop healthier, more marketable rice varieties. This study emphasizes the potential of strategically manipulating starch properties and DF composition to create rice varieties with a lower GI and good CEQ. 

Further research is needed to optimize cooking methods, explore other factors affecting the GI of these rice varieties, and expand the in vivo testing to include a broader range of individuals, including those with pre-existing health conditions. This continued research will contribute to a greater understanding of the complex relationship between rice composition, cooking methods, and human physiology, paving the way for developing more effective strategies for breeding healthier rice varieties for a wide range of consumers. The promising whole-grain soft-texture low- and intermediate-GI lines will be studied to assess their adaptability in farmers’ fields and potential for adoption by farmers.

## Figures and Tables

**Table 1:** Activities on blood sugar GI test day.

Time	Activity	Blood Volume Drawn
7:00 AM	Finger prick blood draw #1 (fasting)	Approximately two drops
7:05 AM	Consume rice sample	No blood draw
7:20 AM	Finger prick blood draw #2 (post-meal)	Approximately two drops
7:35 AM	Finger prick blood draw #3	Approximately two drops
7:50 AM	Finger prick blood draw #4	Approximately two drops
8:05 AM	Finger prick blood draw #5	Approximately two drops
8:35 AM	Finger prick blood draw #6	Approximately two drops
9:35 AM	Finger prick blood draw #7	Approximately two drops

Note: # denotes ‘number’, indicating the sequence of the blood draw.

**Table 2:** The genotyping results of the selected BC_2_F_2_ plants.

Rice Varieties/Lines		wx_5UTR_G/T	ALK_ex8_SNPII_GC/TT	TBGI055578_TC_Chr1	TBGI453598	TBGI454717	TBGI454800	SNP_xa5	Sub1C_loci5	SNP_P100 Xa21	Bph32_4_1223559	OsSTPS2_ATHB-1_TF	OsLecRK3_QBPHR	Aroma_2-3
check	PK+4#20A09	G:G	TT:TT	C:C	C:C	A:A	C:C	T:T	T:T	G:G	C:C	T:T	G:G	Del: Del
check	PK+4#78A03	G:G	GC: GC	T:T	C:C	A:A	C:C	T:T	T:T	G:G	C:C	T:T	G:G	Del: Del
check	PTT1+3	T:T	TT:TT	C:C	T:T	T:T	G:G	T:T	T:T	G:G	C:C	T:T	G:G	Del: Del
BC_2_F_2_	Group 1: 41 plants	G:G	GC: GC	T:T	C:C	A:A	C:C	T:T	T:T	G:G	C:C	T:T	G:G	Del: Del
BC_2_F_2_	Group 2: 8 plants	T:T	GC: GC	T:T	C:C	A:A	C:C	T:T	T:T	G:G	C:C	T:T	G:G	Del: Del
BC_2_F_2_	Group 3: 5 plants	G:G	TT:TT	T:T	C:C	A:A	C:C	T:T	T:T	G:G	C:C	T:T	G:G	Del: Del
BC_2_F_2_	Group 4: 39 plants	T:T	TT:TT	T:T	C:C	A:A	C:C	T:T	T:T	G:G	C:C	T:T	G:G	Del: Del

Note: Plants with stable biotic and abiotic stress resistance genes were categorized into four groups based on functional markers for starch properties (wx_5UTR_G/T for AC and ALK_ex8_SNPII_GC/TT for GT).

**Table 3:** The paste properties of flour from various rice varieties/lines.

Rice Variety/Line	%Starch Content	Pasting Temperature (°C)	Viscosity (RVU)					
			Peak Viscosity	Trough Viscosity	Break Down	Final Viscosity	Setback from Trough *	Setback from Peak **
RD43	77.20 ± 0.20 ^cd^	70.5	171	71	100	126	55	−45
M7881	82.40 ± 0.53 ^a^	71.4	210	59	151	115	56	−95
6D11	83.26 ± 0.64 ^a^	80.4	230	59	171	99	40	−131
9D02	77.85 ± 0.17 ^c^	76.3	212	55	157	94	39	−118
4_E05	76.45 ± 0.17 ^d^	78.6	220	58	162	101	42	−120
12A05	80.70 ± 0.13 ^b^	70.8	233	56	177	101	45	−132
14A03	82.97 ± 0.99 ^a^	77.9	172	71	102	158	87	−15
PK+4#117A08	76.59 ± 0.13 ^d^	68.0	204	116	88	273	157	69

Note: Using a Rapid Visco Analyzer, the flour was determined in a 30 mM silver nitrate solution. Results are the means ± SD on a dry basis and expressed as the whole−grain rice flour percentage. * Setback from trough = final viscosity − trough viscosity. ** Setback from peak = final viscosity − peak viscosity. The letters (a, b, c, d) indicate the results of pairwise comparisons of the means among the different rice varieties/lines.

**Table 4:** In vitro kinetic of starch digestion.

Rice Variety/Line	In Vitro Kinetic of Starch Digestion									
	Freeze Dry (Fine Powder)						Cutting, 0.3 mm			
	Glycemic Index	H90_exp_	H90	GI	Cα	k	Glycemic Index	H90_exp_	H90	GI
Basmati 370	NA						intermediate	23.0 ^d^	30.1 ^f^	63 ^f^
PK+4#20A09	NA						Low	12.9 ^e^	16.0 ^g^	52 ^g^
PK+4#78A03	NA						Low	13.5 ^e^	16.5 ^g^	52 ^g^
RD 43	High	69.3 ^bc^	89.7 ^bc^	111 ^bc^	73.43 ^cd^	0.07 ^ab^	Low	14.4 ^e^	18.68 ^g^	54 ^g^
M7881	High	73.2 ^ab^	88.9 ^bc^	111 ^bc^	75.81 ^abc^	0.06 ^abc^	High	34.0 ^b^	41.28 ^de^	72 ^de^
6D11	High	77.4 ^a^	93.0 ^ab^	114 ^ab^	77.93 ^a^	0.06 ^ab^	High	42.5 ^a^	51.1 ^ab^	80 ^ab^
9D02	High	68.9 ^c^	88.5 ^bc^	110 ^bc^	72.14 ^d^	0.06 ^bc^	High	38.2 ^ab^	49.09 ^bc^	79 ^bc^
4_E05	High	67.6 ^c^	88.4 ^bc^	110 ^bc^	71.41 ^d^	0.05 ^c^	High	42.5 ^a^	55.61 ^a^	84 ^a^
12A05	High	77.0 ^a^	95.4 ^a^	116 ^a^	77.50 ^ab^	0.07 ^a^	High	36.1 ^b^	44.76 ^cd^	75 ^cd^
PK+4#117A08	High	65.8 ^c^	85.9 ^c^	108 ^c^	74.92 ^bc^	0.03 ^d^	intermediate	27.7 ^c^	36.14 ^e^	68 ^e^

Note: The cooked rice was prepared in fine powder and a small-sized cut (0.3 mm). NA = not analyzed. Percentage of total starch hydrolyzed at 90 min (H90) and experiment value (H90exp), equilibrium concentration (Cα), kinetic constant (k), and hydrolysis index (HI), GI = 39.21 + 0.803(H90). GI scale: low GI (GI < 55), intermediate (GI range 56–69), and high GI (GI > 70). Values with different letters in the same column are significantly different with *p* ≤ 0.05.

**Table 5:** Nutritional information of cooked whole-grain rice.

Rice Variety/Line	Energy (kcal)	Protein (g)	Fat (g)	Total Carbohydrate (g)	Dietary Fiber (g)	Available Carbohydrate (g)
PK+4#20A09	124.52 ± 0.75 ^c^	2.57 ± 0.00 ^c^	0.73 ± 0.01 ^d^	27.85 ± 0.02 ^d^	0.94 ± 0.18 ^c^	26.91 ± 0.16 ^b^
PK+4#78A03	125.03 ± 0.25 ^c^	2.78 ± 0.00 ^a^	0.80 ± 0.01 ^c^	27.82 ± 0.01 ^d^	1.14 ± 0.08 ^bc^	26.68 ± 0.08 ^b^
6D11	124.73 ± 0.21 ^c^	2.46 ± 0.01 ^d^	0.84 ± 0.04 ^c^	27.67 ± 0.11 ^e^	1.38 ± 0.10 ^ab^	26.83 ± 0.15 ^b^
9D02	131.71 ± 0.15 ^a^	2.76 ± 0.04 ^a^	0.92 ± 0.01 ^b^	29.24 ± 0.02 ^b^	1.15 ± 0.04 ^bc^	28.09 ± 0.06 ^a^
12A05	131.24 ± 0.68 ^a^	2.63 ± 0.02 ^b^	1.00 ± 0.03 ^a^	29.50 ± 0.04 ^a^	1.57 ± 0.12 ^a^	27.93 ± 0.08 ^a^
2G04	129.40 ± 0.02 ^b^	2.30 ± 0.01 ^e^	0.91 ± 0.02 ^b^	29.05 ± 0.02 ^c^	1.07 ± 0.02 ^c^	27.99 ± 0.03 ^a^

Note: Nutrition values of 50 g cooked whole-grain rice were determined. Values with different letters in the same column are significantly different with *p* ≤ 0.05.

**Table 6:** Nutritional information of rice varieties/lines for in vivo GI test.

Rice Variety/Line	Total Carbohydrate (g)	Protein (g)	Fat (g)	Dietary Fiber (g)	Energy (kcal)	Amount of Cooked Rice (g)
PK+4#20A09	51.74 ± 0.34	4.77 ± 0.03	1.36 ± 0.02	1.74 ± 0.34	231.34 ± 0.05	185.79 ± 1.07
PK+4#78A03	52.14 ± 0.16	5.21 ± 0.01	1.50 ± 0.01	2.14 ± 0.16	234.36 ± 0.19	187.44 ± 0.53
6D11	51.57 ± 0.09	4.58 ± 0.04	1.57 ± 0.09	2.57 ± 0.21	232.43 ± 0.93	186.34 ± 1.06
9D02	52.04 ± 0.07	4.91 ± 0.08	1.64 ± 0.01	2.04 ± 0.07	234.43 ± 0.22	177.99 ± 0.38
12A05	52.81 ± 0.22	4.72 ± 0.03	1.79 ± 0.05	2.81 ± 0.22	234.95 ± 0.55	179.03 ± 0.51
2G04	51.90 ± 0.03	4.11 ± 0.01	1.63 ± 0.03	1.90 ± 0.03	231.16 ± 0.25	178.66 ± 0.24

Note: Data of the cooked whole-grain rice tested each time (g/50 g available carbohydrates).

**Table 7:** In vivo GI values of six rice varieties/lines.

Rice Varieties/Lines	%AC	GI Values ^1,2^		*p*-Values
		Mean ± SD	Classification	
PK+4#20A09	27.60	41.14 ± 10.97	Low	<0.001
PK+4#78A03	27.10	50.47 ± 10.29	Low	<0.001
6D11	12.57	56.11 ± 14.10	Intermediate	<0.001
9D02	15.15	74.35 ± 11.47	High	<0.001
12A05	14.42	53.92 ± 14.87	Low	<0.001
2G04	25.80	41.79 ± 9.54	Low	<0.001

Note: Data represented as mean ± SD. ^1^ Level of GIs were categorized according to high (≥70), medium (56–69), and low (≤55). ^2^ Glucose was used as reference food and defined as 100. *p*-values < 0.05 were considered a statistically significant difference in GI between the rice samples and the reference food.

**Table 8:** The SDF and IDF content of rice varieties/lines.

Rice Variety/Line	SDF	IDF	SDF–IDF
PK+4#20A09	0.27 ± 0.02 ^c^	2.71 ± 0.54 ^b^	0.10 ^b^
PK+4#78A03	0.71 ± 0.00 ^b^	2.86 ± 0.25 ^ab^	0.25 ^ab^
6D11	0.65 ± 0.06 ^b^	3.65 ± 0.38 ^a^	0.18 ^ab^
9D02	0.25 ± 0.05 ^c^	3.18 ± 0.07 ^ab^	0.08 ^b^
12A05	1.15 ± 0.01 ^a^	3.51 ± 0.34 ^a^	0.33 ^a^
2G04	0.77 ± 0.41 ^b^	2.49 ± 0.36 ^b^	0.33 ^a^

Note: The candidate rice lines and PK+4 varieties 20A09 and 78A03 were tested. Results are the mean ± SD on a dry basis and expressed as the percentage of whole grain flour. Values with different letters in the same column significantly differ with *p* ≤ 0.05.

## Data Availability

The original contributions presented in the study are included in the article/Supplementary Materials, further inquiries can be directed to the corresponding author.

## Supplementary Materials

The following supporting information can be downloaded at https://www.mdpi.com/article/10.3390/foods13223627/s1: Table S1: The data on days to flowering, days to maturity, and yield (kg per ha) of the BC_2_F_4_ rice population; Table S2: Amylose content and rapidly available glucose (RAG) of cooked rice with low gelatinization temperature (GT); Table S3: Amylose content and rapidly available glucose (RAG) of cooked rice with high gelatinization temperature (GT); Table S4: Amylose content, rapidly available glucose, and slowly available glucose of cooked rice with low and high gelatinization temperature; Figure S1: The whole-grain rice samples were cooked using a rice cooker with a rice-to-water ratio 1:2 and cooked for 30 to 40 min; Figure S2: Days to flowering, days to maturity, and yield of BC_2_F_3_ population.
