# Supplementary material for: Development of Whole-Grain Rice Lines Exhibiting Low and Intermediate Glycemic Index with Decreased Amylose Content
Source: Foods. 2024 Nov 14;13(22):3627. doi: 10.3390/foods13223627 (PMC11593259; doi:10.3390/foods13223627)
Supplement: Supplementary file 1 [file foods-13-03627-s001.zip › foods-3306561-supplementary.pdf]

Supplementary Materials

Supplementary Table S1 shows the data on days to flowering, days to maturity, and yield (kg per ha) of the BC_2F_4 rice population.

Line No.	Day to Flowering (Days)	Day to Maturity (days)	Yield (Kg per ha)
10D04	112	135	4335
15B12	123	150	4271
14H08	121	139	3959
10E.02	127	153	3635
2G04	128	153	3527
6C11	110	131	3484
12A05	112	133	3360
8C12	118	146	3339
3C03	118	147	3325
14B10	110	139	3295
2D09	119	146	3256
8D10	122	150	3112
3E.05	112	139	3103
14F12	112	143	3090
15C05	119	146	3031
14A12	113	135	3013
15D02	116	146	2997
14A08	129	154	2865
9B09	112	135	2855
14E.01	114	133	2756
14E.12	110	139	2737
13A06	108	133	2604
10B09	127	151	2598
8G04	108	135	2551
9G04	121	149	2548
9D06	118	150	2543
7A02	112	135	2441
16B09	120	150	2408
14A04	123	153	2404
10A11	122	149	2387
14B01	113	135	2327
16H02	113	135	2316
14H03	131	156	2239
14A03	131	156	2187

Line No.	Day to Flowering (Days)	Day to Maturity (days)	Yield (Kg per ha)
10C01	122	153	2156
12D01	127	153	2099
10A01	118	133	2035
5B07	110	135	2025
9G01	123	153	1907
10G01	128	153	1863
15F02	112	131	1859
6D11	112	146	1853
4D02	112	139	1811
16E.03	116	145	1789
2G03	131	156	1726
5D03	135	158	1682
9E.03	108	133	1661
14F10	108	131	1648
7F09	133	156	1512
3E.07	127	153	1499
12B01	122	153	1334
8F01	106	131	1319
5B09	116	153	1280
8F03	110	131	1277
9B08	112	146	1064
8B12	135	156	933
9A04	133	156	909
5H03	108	139	847
9D02	127	156	677
5B06	102	127	673
5G06	99	129	614
4E.05	113	139	348

Supplementary Table S2 Amylose content and rapidly available glucose (RAG) of cooked rice with low gelatinization temperature (GT)

Rice lines	GT	%Amylose content (base on rice flour)	Rapidly available glucose at 20 mins base on dry solid of cooked rice
5B07	Low	10.52	88.0 ± 2.1a
9E03	Low	11.57	84.7 ± 1.0abc
16H02	Low	11.79	84.6 ± 1.3abc
5B06	Low	11.91	83.0 ± 3.4bcd
7A02	Low	12.57	81.7 ± 1.8bcdef
8F01	Low	12.59	84.1 ± 1.5bcd
14E01	Low	12.79	73.2 ± 2.7jkl
5G06	Low	13.05	71.5 ± 2.0klm
9A04	Low	13.08	77.4 ± 2.8ghi
14B01	Low	13.25	73.3 ± 1.4jkl
9B09	Low	13.69	71.2 ± 0.5klm
14A12	Low	13.86	69.2 ± 2.5 m
2D09	Low	13.88	78.7 ± 3.1fgh
14E02	Low	14.03	70.3 ± 2.0lm
12A05	Low	14.42	64.6 ± 3.3n
10A01	Low	14.46	79.2 ± 0.4efgh
14B10	Low	14.66	81.1 ± 1.1cdef
8G04	Low	14.76	80.6 ± 1.5defg
5H03	Low	14.88	82.6 ± 0.1bcde
10D04	Low	15.36	74.0 ± 0.9ijk
8B12	Low	15.61	72.3 ± 0.8klm
14H08	Low	15.78	82.9 ± 1.1bcd
6C11	Low	16.12	85.1 ± 1.5ab
13A06	Low	16.28	81.6 ± 2.3bcdef
4D02	Low	16.69	76.0 ± 0.3hij
15C05	Low	16.77	84.1 ± 1.3bcd
3E05	Low	19.26	69.7 ± 1.4lm
8C12	Low	19.50	82.3 ± 2.3bcde
8F03	Low	22.69	42.9 ± 0.6o
15D02	Low	24.37	37.8 ± 0.9pq
16B09	Low	25.34	26.8 ± 1.6r
16E03	Low	25.54	27.1 ± 0.6r
10B09	Low	26.02	36.2 ± 1.5q
117A08	Low	26.80	40.3 ± 1.6op

Results are the mean ± SD on a dry basis and expressed as the percentage of whole grain flour. Values with different letters in the same column significantly differ with $p \leq 0.05$.

Supplementary Table S3 Amylose content and rapidly available glucose (RAG) of cooked rice with high gelatinization temperature (GT)

Rice lines	GT	%Amylose content (base on rice flour)	Rapidly available glucose at 20 mins base on dry solid of cooked rice
6D11	High	12.57	79.6 ± 1.5a
4E05	High	13.61	58.5 ± 3.4b
9D02	High	15.15	60.9 ± 1.0b
14F10	High	21.55	31.5 ± 0.0hi
9G01	High	23.25	26.0 ± 0.0jklm
12B01	High	23.71	33.7 ± 2.5fgh
14A08	High	23.93	37.7 ± 1.2e
3E07	High	24.37	26.6 ± 1.4jkl
14F12	High	24.42	33.2 ± 2.3fgh
3C03	High	24.66	28.8 ± 0.5ij
12D01	High	24.88	29.5 ± 0.2ij
14A03	High	25.00	42.4 ± 0.1d
9D06	High	25.61	36.5 ± 3.0ef
2G04	High	25.80	33.2 ± 0.2fgh
14H03	High	25.92	34.6 ± 2.5efgh
10A11	High	26.02	25.0 ± 1.7klm
9B04	High	26.34	28.5 ± 0.3ijk
5D03	High	26.46	29.4 ± 2.0ij
5B09	High	26.61	43.7 ± 0.3d
9B08	High	26.63	31.7 ± 3.5hi
15F02	High	26.80	43.9 ± 0.1d
10G01	High	26.87	49.3 ± 0.4c
14A04	High	27.07	35.4 ± 1.0efg
10C01	High	27.77	44.7 ± 2.6d
10E02	High	28.21	31.9 ± 3.3ghi
2G03	High	28.55	22.9 ± 2.3m
15B12	High	29.22	29.0 ± 0.6ij
8D10	High	29.63	44.8 ± 0.5d
7F09	High	29.74	23.6 ± 1.6lm

Results are the mean ± SD on a dry basis and expressed as the percentage of whole grain flour. Values with different letters in the same column significantly differ with $p \le 0.05$.

Supplementary Table S4 Amylose content, rapidly available glucose, and slowly available glucose of cooked rice with low and high Gelatinization temperature

Rice lines	GT	%Amylose content (base on rice flour)	available glucose base on dry solid of cooked rice	
			Rapid available glucose at 20 mins	Slow available glucose at 120 mins
12A05	Low	17.61	64.6 ± 3.3b	18.8 ± 0.0f
117A08	Low	28.86	40.3 ± 1.6de	36.1 ± 1.6d
8F03	Low	30.31	42.9 ± 0.6d	45.6 ± 0.6ab
15D02	Low	31.29	37.8 ± 0.9e	48.1 ± 1.1a
6D11	High	15.56	79.6 ± 1.5a	13.5 ± 1.3g
9D02	High	16.97	60.9 ± 1.0c	26.2 ± 1.6e
4_E05	High	17.42	58.5 ± 3.4c	26.1 ± 2.9e
9G01	High	29.28	26.0 ± 0.0i	41.6 ± 2.8c
12B01	High	29.57	33.7 ± 2.5f	43.0 ± 2.5bc
12D01	High	29.72	29.5 ± 0.2gh	41.1 ± 1.8c
14A08	High	29.92	37.7 ± 1.2e	43.6 ± 1.7bc
14A03	High	29.97	42.4 ± 0.1d	35.6 ± 3.0d
3C03	High	30.23	28.8 ± 0.5ghi	43.6 ± 1.0bc
14F12	High	30.31	33.2 ± 2.3f	42.3 ± 1.5bc
3_E07	High	30.34	26.6 ± 1.4hi	43.8 ± 0.6bc
14F10	High	30.74	31.5 ± 0.0gh	43.3 ± 1.3bc

Results are the mean ± SD on a dry basis and expressed as the percentage of whole grain flour. Values with different letters in the same column significantly differ with $p \leq 0.05$.

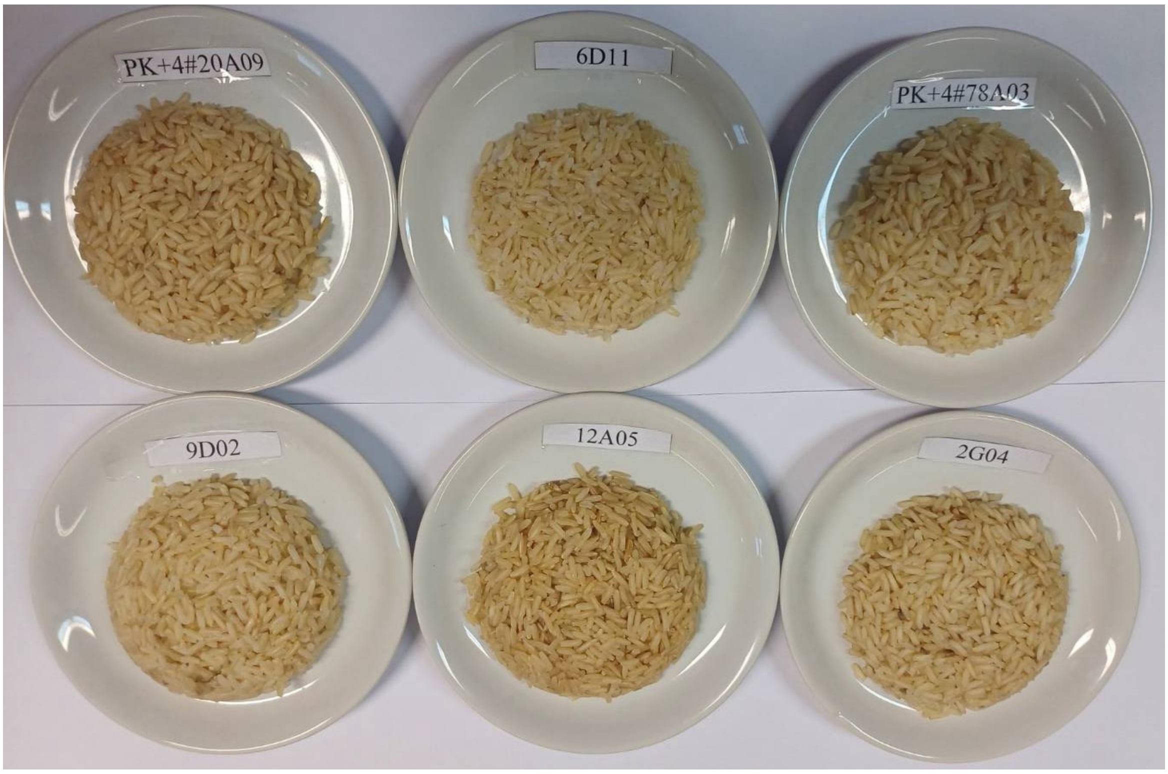

Supplementary Figure S1 The whole-grain rice samples were cooked using a rice cooker with a rice-to-water ratio 1:2 and cooked for 30 to 40 minutes.

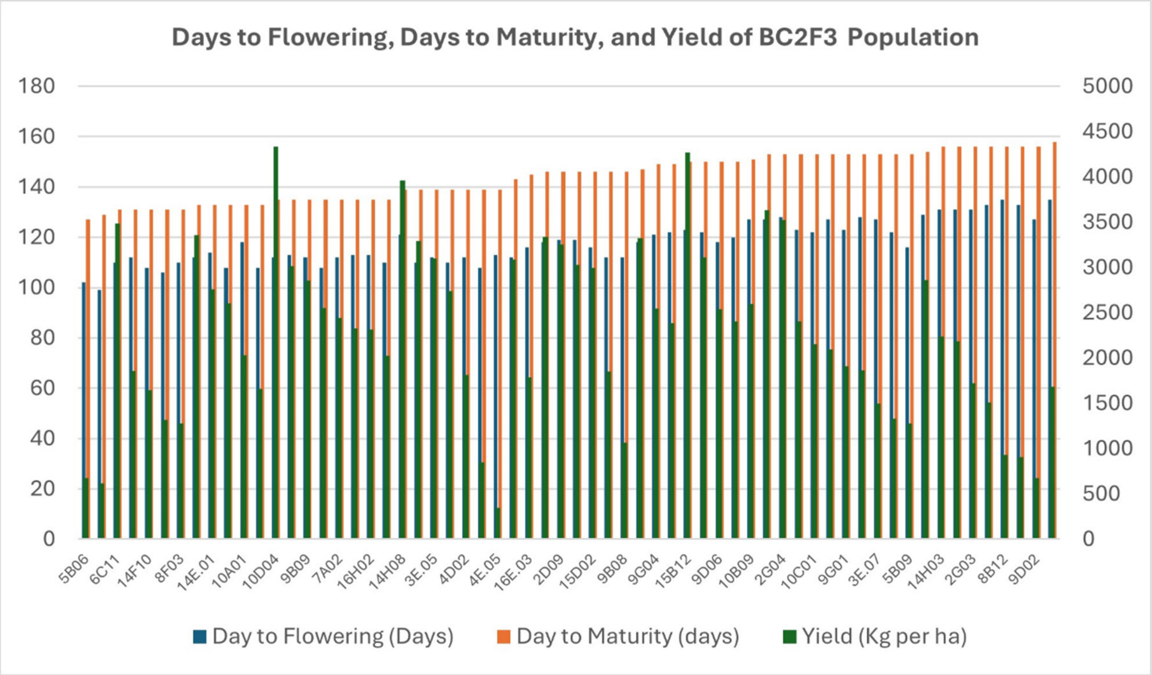

Supplementary Figure S2 Days to Flowering, Days to Maturity, and Yield of BC_2F_3 population.

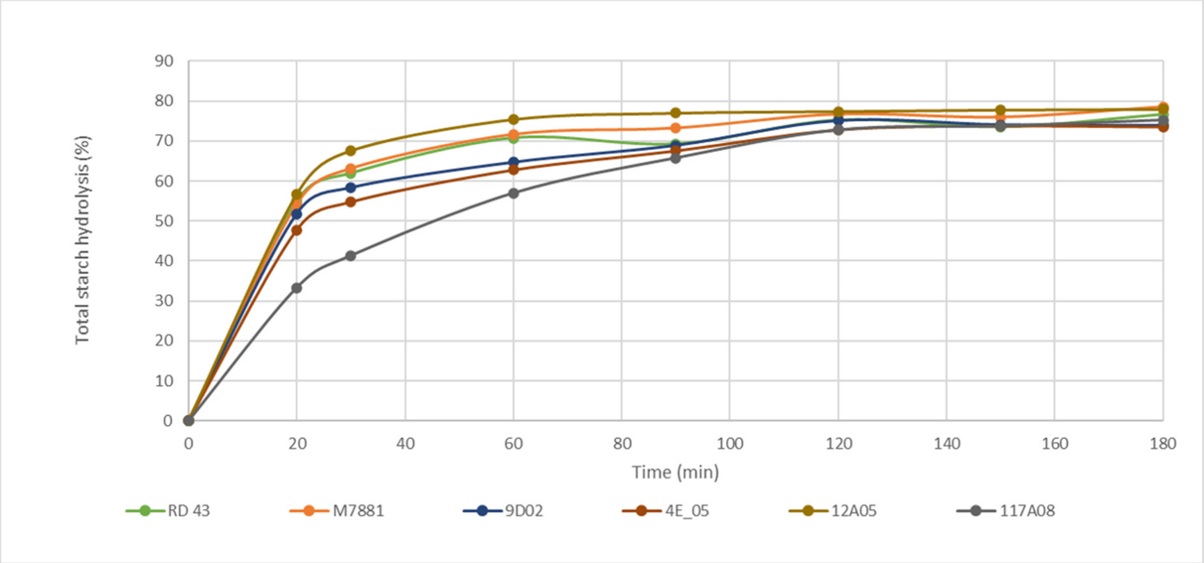

Supplementary Figure S3 The rate of starch digestion by amylase enzyme over varying incubation times (20 to 180 minutes) differs among cooked whole-grain rice varieties. The samples was prepared as freeze-dried (fine powder).
